# Identification of factors associated with diagnostic error in primary care

**DOI:** 10.1186/1471-2296-15-92

**Published:** 2014-05-12

**Authors:** Sergio Minué, Clara Bermúdez-Tamayo, Alberto Fernández, José Jesús Martín-Martín, Vivian Benítez, Miguel Melguizo, Araceli Caro, María José Orgaz, Miguel Angel Prados, José Enrique Díaz, Rafael Montoro

**Affiliations:** 1 Andalusian School of Public Health, Cuesta del Observatorio 4, Campus Universitario de Cartuja, 18080 Granada, Spain; 2 Institute de recherche en santé publique. Université de Montréal, Montréal, Canada; 3 CIBERESP. CIBER de Epidemiología y Salud Pública, Madrid, Spain; 4 Universidad de Granada, Facultad de Ciencias Económicas y Empresariales, Campus Universitario de la Cartuja, 18011 Granada, Spain; 5 Centro de salud Almanjayar. Servicio Andaluz de Salud, C/Pintor Joaquín Capulino Jaúregui SN., 18013 Granada, Spain; 6 Centro de salud Cartuja. Servicio Andaluz de Salud, C/Pintor Joaquín Capulino Jaúregui SN., 18013 Granada, Spain; 7 Centro de salud Gran Capitán. Servicio Andaluz de Salud, C/Gran Capitán, 10, 18002 Granada, España; 8 Distrito de Atención Primaria de Granada -Metropolitano. Servicio Andaluz de Salud, Calle Doctor Azpitarte, 4, 18012 Granada, Spain; 9 Département d’administration de la Santé, École de santé publique, 7101, Avenue du Parc., H3N 1X9 Montréal, Québec, Canada

**Keywords:** Primary care, Diagnostic errors, Decision-making

## Abstract

**Background:**

Missed, delayed or incorrect diagnoses are considered to be diagnostic errors. The aim of this paper is to describe the methodology of a study to analyse cognitive aspects of the process by which primary care (PC) physicians diagnose dyspnoea. It examines the possible links between the use of heuristics, suboptimal cognitive acts and diagnostic errors, using Reason’s taxonomy of human error (slips, lapses, mistakes and violations). The influence of situational factors (professional experience, perceived overwork and fatigue) is also analysed.

**Methods:**

Cohort study of new episodes of dyspnoea in patients receiving care from family physicians and residents at PC centres in Granada (Spain). With an initial expected diagnostic error rate of 20%, and a sampling error of 3%, 384 episodes of dyspnoea are calculated to be required. In addition to filling out the electronic medical record of the patients attended, each physician fills out 2 specially designed questionnaires about the diagnostic process performed in each case of dyspnoea. The first questionnaire includes questions on the physician’s initial diagnostic impression, the 3 most likely diagnoses (in order of likelihood), and the diagnosis reached after the initial medical history and physical examination. It also includes items on the physicians’ perceived overwork and fatigue during patient care. The second questionnaire records the confirmed diagnosis once it is reached. The complete diagnostic process is peer-reviewed to identify and classify the diagnostic errors. The possible use of heuristics of representativeness, availability, and anchoring and adjustment in each diagnostic process is also analysed. Each audit is reviewed with the physician responsible for the diagnostic process. Finally, logistic regression models are used to determine if there are differences in the diagnostic error variables based on the heuristics identified.

**Discussion:**

This work sets out a new approach to studying the diagnostic decision-making process in PC, taking advantage of new technologies which allow immediate recording of the decision-making process.

## Background

### Diagnostic error

Missed, delayed or incorrect diagnoses are considered to be diagnostic errors [1]. Although the incidence of diagnostic error is difficult to establish [2], it is estimated to be between 5% and 20% [3], depending on the medical speciality analysed. Berner and Graber [4] distinguish between “perceptual” specialities (radiology and anatomical pathology), where the diagnosis is made based on the perception of an image, and other clinical fields (family medicine, internal medicine, emergency department). The incidence of diagnostic error in the former is between 2% and 5%, while the incidence in the latter can be up to 15% [5]. In the United States, it is estimated that more than 150,000 patients per year may undergo misdiagnosis-related harm, and 50,000 missed diagnostic opportunities occur each year in primary care (PC) alone [6]. Diagnostic errors lead to more deaths than any other medical error type [7], and this can have major legal repercussions: diagnostic errors are the leading cause of malpractice claims in the United States [8].

However, few studies on the diagnostic process have been published, probably because it is still considered to be more of an individual art than a science. The complexity of the diagnostic process and the lack of established methods for its analysis also play a part. There are even fewer studies on the diagnostic process in the PC setting [9].

### Clinical reasoning

In order to provide the right clinical care, a correct diagnosis is essential, although not sufficient on its own. To reach the right diagnosis, physicians must take a complete medical history and perform the right physical examinations and further tests. Not ordering the correct tests is potentially just as dangerous as performing unnecessary tests, which can lead to iatrogenic effects and overdiagnosis [10].

Studies on decision-making under conditions of uncertainty in the fields of psychology and behavioural economics have very slowly started to spread into the medical field. Stanovich and West [11] coined the terms “system 1” and “system 2” to refer to the 2 systems of cognitive function. System 1 operates automatically and quickly, with little or no effort and no sense of voluntary control. System 2 allocates attention to the effortful mental activities that demand it, and its operations are often associated with the subjective experience of agency, choice and concentration. Diagnostic error is typically viewed as a cognitive failing [12], often caused by biases linked to system 1 (non-analytical, intuitive thinking). Some authors [13] believe that these cognitive biases can be a warning sign for possible diagnostic errors.

Heuristics are “the strategies that people use deliberately in order to simplify judgemental tasks that would otherwise be too difficult for the typical human mind to solve” [14]. Kahneman, Slovic and Tversky [15] identified different types of heuristics involved in the decision-making process. However, not all researchers believe that the use of heuristics (and by extension, system 1) in clinical reasoning necessarily leads to a higher risk of error [16].

This study uses only the 3 general heuristics described initially by Kahneman and Tversky, the validity of which has been confirmed by many empirical findings over the last few decades [17]: 1) Availability, i.e. the diagnostic alternatives available during the reflective process at a particular point in medical care; 2) Representativeness, i.e. the case’s similarity to a particular known diagnostic category; and 3) Anchoring and adjustment, i.e. how well the information generated during the diagnostic process fits with the initial diagnostic hypothesis.

“Suboptimal cognitive acts” (SCAs) are cognitive errors that deviate from the optimal cognitive process, and which could lead to an adverse event [18]. Based on Reason’s model of unsafe acts [19], cognitive errors can occur as a result of 2 types of action: unintentional and intentional.

1) Unintentional actions: these occur when routine activities are inadequately performed because of failings in attention (slips) or memory (lapses). In these cases, the error is caused by a failure in the storage and/or execution stage in the action sequence, regardless of whether the action is appropriate or not. It is therefore largely influenced by working conditions, especially overwork and fatigue.

2) Intentional actions: deliberate actions that may lead to an error because the person lacks the knowledge required to tackle the situation (mistakes), or because the person disregards the appropriate procedure (violations). In these cases, the error is due to a lack of knowledge (in the first case) or compliance (in the second). They are therefore influenced by experience and attitude.

### Factors associated with the diagnostic process in PC

PC physicians work under vastly different conditions from their colleagues in emergency or hospital departments. In PC settings, patients usually present with poorly defined, often psychological or social symptoms. Some patients attend in the initial stages of the disease, while others have confirmed diagnoses. PC physicians see patients whose problems have not yet been reduced to a specific category, so there is a very broad range of possible diagnoses [20]. In order to tackle this diverse range of problems appropriately, PC must fulfil 4 essential criteria [21]: accessibility, comprehensiveness of services, coordination of care within the healthcare system, and longitudinality (the capacity to provide continuous (ongoing) care throughout a patient’s lifetime). In addition to these specific aspects, the diagnostic process in PC is determined by factors that influence any care context: professional experience, overwork, fatigue and stress [18,22]. The increase in PC activities often leads to overwork, and this can have a direct influence on the diagnostic process.

Studying the diagnostic process in PC requires an analysis of the complete process a physician follows when faced with symptoms, not diseases, from the moment care starts to the moment a diagnosis is reached. Dyspnoea was selected for this study because it is a frequent PC problem and provides an opportunity for a differential diagnostic procedure.

The aims of this study are to:

1) Identify and describe the SCAs that occur during care of patients with dyspnoea attending PC centres.

2) Analyse cognitive aspects of the process by which PC physicians diagnose dyspnoea using heuristics.

3) Analyse the relationship between situational factors (perceived overwork, fatigue, professional experience) and SCAs during the diagnostic process in patients with dyspnoea in PC settings.

4) Analyse the relationship between those situational factors and the use of heuristics during the diagnostic process in patients with dyspnoea in PC settings.

## Methods

### Study design

**Figure 1:**
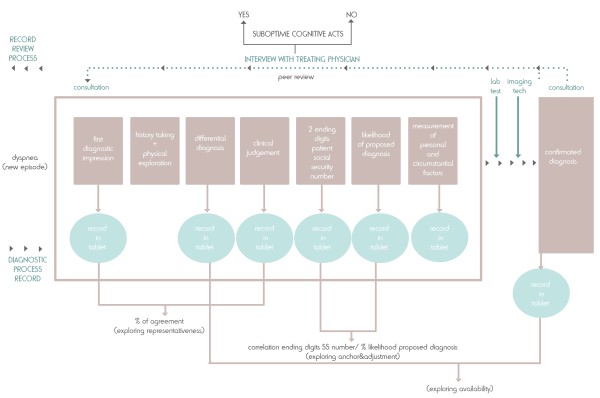
**Study procedure.** New episodes of dyspnea and identification of suboptimal cognitive acts.

We designed a prospective cohort study of new episodes of dyspnoea. The cohort monitoring period begins when the patient first attends because of a new episode and ends when a concrete diagnosis is reached. This period is estimated to be between 2 days and 8 months [18]. The study procedure is shown in Figure 1.

### Selection of centres

We selected 4 PC centres in the province of Granada (Spain). The family physicians working at the centres have different levels of experience, the centres have different patient numbers, and the patients come from different socioeconomic backgrounds (lower, middle-lower, middle-upper and upper classes). 2 distinct physician types were selected: residents and experienced family physicians.

### Study subjects

The study analyses diagnostic processes in patients registered at the practices of the participating family physicians, attending because of a new episode of dyspnoea. A consecutive sampling method was used, recruiting diagnostic procedures from the accessible population of patients attending because of a new episode of dyspnoea.

### Sample size

We calculated the number of cases required based on the method set out by Zwaan et al. [18]. In that study, in order to obtain a substantial number of SCAs in each of the 5 categories (slips, lapses, mistakes, violations, patient record problems), a confidence interval was calculated assuming an equal distribution of suboptimal cognitive acts in each category (20% of SCAs in each category). An average of 1.5 SCAs was expected per patient record, so 250 records of dyspnoea (375/1.5) were required. Based on this, the number of cases of dyspnoea expected in our study is 384.

### Variables

As independent variables, we are going to explore factors related with the patient, the setting and the professional. The dependent variables are those related with the errors. These are described in Table 1.

**Table 1:** Variables studied

**Independent variables**			**Dependent variables**	
**Physician**	**Setting**	**Patient**	**Suboptimal cognitive acts**	**Use of heuristics**
Age/Sex	Number of patients on list	Age/Sex	Lapses	Representativeness
Years of experience	Number of patients attended	Nationality	Slips	Availability
Speciality	Type of consultation	Profession	Mistakes	Anchoring and adjustment
CME related to dyspnoea	Socioeconomic background	Education	Violations	
Career level	Hospital referral	Employment		
Workload	Access to electronic medical record during physician-patient encounter	Morbidity		
Fatigue				

### Study period

The study period deemed necessary to recruit the number of patients required and carry out the diagnostic process is 1 year. The audit is carried out at the end of each diagnostic process.

### Data collection

The data are collected in the following phases and through the following sources:

#### PHASE 1. Record of the diagnostic process (provided by the physician)

1. *Data from the physician*

Once they have agreed to take part in the project, the PC physicians fill out records detailing variables about themselves (listed in the first column of Table 1). They also fill out the patients’ electronic clinical records in the software application used by all healthcare professionals working for the Andalusian Health Service, the region’s public health service.

2. *Data from the diagnostic process*

For each patient attending with a new episode of dyspnoea, the physicians fill out 2 questionnaires via a tablet computer with an internet connection. The questionnaire is available as a wufoo application (http://www.wufoo.com) saved on the tablet’s desktop. Tables 2 and 3 describes the questionnaires used.

**Table 2:** Questionnaire evaluation of first diagnosis

**Variable**	**Headland**	**When it is filled out**	**Explanation**
1. Evaluation Of first diagnosis			
Diagnosis	First diagnostic impression	After reason for consultation described	Possible use of representativeness heuristic (“Of which disease is this specific episode of dyspnoea representative?”)
	Differential diagnosis (3 possible diagnoses identified in order of likelihood)	After the medical history is taken and physical examination is performed	Possible use of availability heuristic (diagnostic possibilities that come to the physician’s mind at the time of diagnosis)
	Diagnosis	After the differential diagnosis	
	Last 2 digits of patient’s social security number	After diagnosis	Possible use of anchoring and adjustment heuristic in estimation of the likelihood of the diagnosis being correct
	Likelihood that the proposed diagnosis is correct (%)	After recording the last 2 digits of the patient’s social security number	
Context	Subjective workload	During consultation	Identification of specific situational factors during the consultation that could influence the diagnostic process, in terms of both use of heuristics and performance of suboptimal cognitive acts/errors
	Perceived subjective mental workload (adapted from NASA-TLX	During consultation	Evaluation of aspects of mental demand, physical demand, performance, effort and frustration
	Adaptation of the Spanish version of the Swedish Occupational Fatigue Inventory (SOFI)	During consultation	Evaluation of work-related fatigue
	**Characteristics of the encounter between physician and patient:**	During consultation	Characteristics of the encounter
	• Consultation type		
	• Number of patients attended		
	• Patient’s number in the order of patients attended		
	• Consultation delay		
	**Relationship with the patient over time:**	During consultation	Longitudinality (capacity to provide care to patients over time)
	• Time physician has been providing care for the patient		
	• Time physician has been providing care for the same patient list		
	• Number of previous visits by the patient in the last year		
	• Date of last visit		

**Table 3:** Questionnaire of final diagnosis and audit

**Variable**	**Headland**	**When it is filled out**	**Explanation**
1. Evaluation Of final diagnosis			
	- Final diagnosis confirmed	When final diagnosis of underlying cause of dyspnoea is made	Possible use of availability heuristic (diagnostic possibilities that come to the physician’s mind at the time of diagnosis) when the hypotheses are compared with the final diagnosis
	- Time since first visit		
	- Number of visits		
2. AUDIT			
Medical history	Personal and family history	After diagnosis is confirmed	Evaluation of accuracy of diagnosis
	Characteristics of dyspnoea		
	Accompanying symptoms		
Physical examination			
Further tests			
Appropriateness of diagnostic process			
Performance of suboptimal cognitive acts	Identification of error type (slip, lapse, mistake, violation)		Evaluation of existence of suboptimal cognitive act
Identification of diagnostic error	Stage in which it occurs		Evaluation of the existence of an error and its consequences
	Existence of misdiagnosis-related damage and damage type		
	Possibility of damage prevention		

### PHASE 2. Review of the diagnostic process (provided by the evaluators)

A. *Identification of the optimal diagnostic process*

The methodology used by Zwaan [23] has been used as a reference for the audit and adapted to the PC setting.

The clinical practice guidelines set out by the Spanish Society of Family and Community Medicine (semFYC) were reviewed. As no clinical guidelines on the management of dyspnoea in PC were found, an exhaustive review of the literature on dyspnoea care in PC was carried out. The dyspnoea care procedure in PC was revised based on the articles found.

These reviewed guidelines were sent to 4 family physicians with more than 20 years of clinical experience, who made all the necessary corrections to create a consensus reference document.

B. *Creation of the audit questionnaire*

Based on the consensus document created, a diagnostic process audit questionnaire [23] analysing the following 7 phases of the process was created: 1) medical history; 2) physical examination; 3) laboratory test results; 4) scans; 5) diagnosis; 6) treatment; 7) follow-up.

The questionnaire was revised and piloted by the participants in the audit process, and a second questionnaire was created based on the suggestions made after the pilot.

The final audit questionnaire will be filled out via wufoo when the audit is carried out.

C. *Audit*

Each of the cases will be assessed by 2 physicians (not including the physician who performs the diagnosis). If the evaluations of the 2 physicians are different, the case will be assessed by a third evaluator. The physician-evaluators have accredited clinical care experience and have been trained in how to use the audit guide. The stages of data collection will be analysed based on two criteria: 1) whether the correct information was collected; and 2) whether the information was interpreted correctly.

Variables associated with the possible use of heuristics, the performance of suboptimal cognitive acts and the possible existence of diagnostic error will be collected.

D. *Review of the diagnostic process with the physician in charge of the patient’s care*

The diagnostic process audit will be reviewed by the evaluators and the physicians who performed the diagnostic process through an interview in order to analyse any cognitive acts that took place, as well as any possible diagnostic errors.

#### PHASE 3. Review of centre reports, to obtain information about centre characteristics

### Data analysis

A descriptive statistical analysis of diagnostic errors, suboptimal cognitive acts and use of representativeness, availability and anchoring heuristics will be carried out.

A bivariate analysis will be performed to establish if there are any differences in the diagnostic error variables based on the heuristics identified and the independent variables. To do this, a Chi-square or Fisher’s exact test will be used for samples with fewer than 5 individuals.

Multivariate models will be used to determine if there are any differences in the diagnostic error variables based on the heuristics identified, adjusting for the other independent variables, using logistic regression models.

### Confidentiality and ethical approval

This project has been approved by the Regional Research Ethics Committee of the province of Granada (Spain).

All participating patients agreed to take part in the research project through the corresponding informed consent process.

All participating physicians also signed consent forms in order to take part, expressly agreeing to keep all the information collected confidential. None of the data collected could be used to identify patients. ID numbers only were used.

The database created, using information exclusively from the questionnaires available through wufoo, was not linked in any way to the electronic medical records of the patients studied.

## Discussion

### Strengths

The empirical evidence gathered over the last few decades about cognitive biases and the use of heuristics in decision-making under conditions of uncertainty [17] has not yet been satisfactorily transferred to the medical field. In fact, there are major differences of opinion about the role of heuristics in clinical decision-making and the effectiveness and risks of their use [24,25].

Furthermore, some recent reviews [26,27] warn of the lack of studies analysing the relationship between the reasoning process and diagnostic errors, despite the relevance of the link. This lack of empirical research is particularly pronounced in PC, even though it is the setting where most clinical encounters take place and where the clinical decision-making process is performed under the greatest conditions of uncertainty. Some authors [28] believe that the next step in diagnostic error research would be to better understand how hypotheses developed under experimental conditions translate into real-world situations. Despite its methodological limitations, our study could lead to interesting conclusions to better understand the diagnostic process in real-world PC settings, especially with regard to its determining factors, both situational (overwork, fatigue, stress) or experience-related (training, years of experience).

It can be difficult to collect information about the clinical reasoning process at the time it takes place. However, this can be overcome through the use of recently developed and widely used information technologies in the form of hardware (tablets) and software (apps with links to websites that collect data).

The methodology proposed allows the diagnostic process as a whole to be assessed, using both prospective and retrospective sources [18]. One major strength of the method is the involvement of the participating physicians themselves, particularly the physician being assessed in the interview analysing his/her cognitive processes. This approach makes it possible to check, adjust and increase the information available to the evaluators, turning the audit into a process of ongoing improvement of the physician’s practice.

### Limitations

The study is affected by volunteer selection bias, the Hawthorne effect, and the selection criteria used (only cases described as dyspnoea by the physician at the time of consultation are included, excluding false negatives). There is also a differential classification bias, in the sense that it is known that diagnostic error will be underestimated for several reasons: the physician already knows which symptom is being studied, is aware that he/she is going to be observed, and over time there may be a learning effect in the care of patients with this symptom.

There are no objective criteria defining the optimal diagnostic process in patients with dyspnoea attended by PC physicians, so there is no gold standard with which the processes performed can be compared. However, in order to have a reference with which to compare the diagnostic processes performed, we followed the procedure used in other similar studies [18]: review of the best scientific evidence available, review and consensus by expert professionals, and design, pilot and application of a questionnaire for the audit.

Given the study period used in the analysis, it is possible that in some cases the diagnostic process may be deemed finished when it is not, and this means that certain errors may not be detected. There are 3 main causes of losses during follow-up: a) leaving the study; b) death by another cause; and c) “administrative losses” caused by early termination of the study due to reasons other than those initially predicted. To reduce this bias, all of the information available about the lost cases will be collected in order to have as much data as possible about the losses, to quantify their causes and thus evaluate the validity of the study.

Widely available, easy-to-use, uncomplicated electronic devices have been used to facilitate recording of the cognitive and diagnostic processes. Each physician uses a tablet with a 3G connection to record the information in the health centres and in patients’ homes. By storing the data on a website as soon as it is recorded, it is immediately available for analysis. However, the possibility of under-recording and delays in the recording process cannot be ruled out, and these could limit the validity of the results obtained.

The medical history review process may also be subject to hindsight bias, i.e. analysis of the diagnostic processes may be affected by prior knowledge of the final result.

### Trial status

The medical history audit is currently being carried out.

### Conclusions

This paper sets out a methodology for studying the diagnostic process in PC adapted from previous works by Zwaan et al. [18,23]. It also takes advantage of the possibilities that new technologies offer in terms of immediate recording of the clinical decision-making process, in order to assess the possible use of heuristics (representativeness, availability and anchoring) during the diagnostic process. Reason’s taxonomy, which categorises actions into unintentional (slips and lapses) and intentional ones (mistakes and infractions), is used as a reference for identifying possible suboptimal cognitive acts that physicians may have performed during the dyspnoea diagnostic process. Discussing the results obtained in the audit with each physician will facilitate learning about the diagnostic process, increasing knowledge of the key factors determining clinical reasoning.

The methodology also makes it possible to analyse the influence of clinical experience and situational factors (overwork, fatigue, stress) on the diagnostic process.

## Abbreviations

PC: Primary care; SCAs: Suboptimal cognitive acts; semFYC: Spanish society of family and community medicine.

## Competing interests

The authors declare that they have no competing interests.

## Authors’ contributions

SM directed the study design process, planned the review and adaptation, drafted the manuscript and wrote the final version. CB participated in the study conception and design, revised the draft manuscript and critically revised the manuscript. AF participated in the study conception and design, revised the draft manuscript and critically revised the manuscript. JJM supervised the study protocol and its design, and critically revised the final manuscript. VB performed the bibliographical review and participated in the design of the audit. MM participated in the study conception, reviewed the design and implementation of the audit, and participated in the design of the dyspnoea protocol and the evaluation questionnaire. AC participated in the study design. MJO reviewed the design and implementation of the audit, and participated in the design of the dyspnoea protocol and the evaluation questionnaire. MAP reviewed the design and implementation of the audit, and participated in the design of the dyspnoea protocol and the evaluation questionnaire. JED reviewed the design and implementation of the audit, and participated in the design of the dyspnoea protocol and the evaluation questionnaire. RM reviewed the design of the initial project. All the authors revised the final version of the manuscript. All authors read and approved the final manuscript.

## Pre-publication history

The pre-publication history for this paper can be accessed here:

http://www.biomedcentral.com/1471-2296/15/92/prepub

## Previous presentations

The approach used in this project was presented as a poster presentation at the Diagnostic Error in Medicine 6th International Conference held in Chicago (2013).
